# Supplementary figures and images for: The complex architecture and epigenomic impact of plant T-DNA insertions
Source: PLoS Genet. 2019 Jan 18;15(1):e1007819. doi: 10.1371/journal.pgen.1007819 (PMC6338467; doi:10.1371/journal.pgen.1007819)

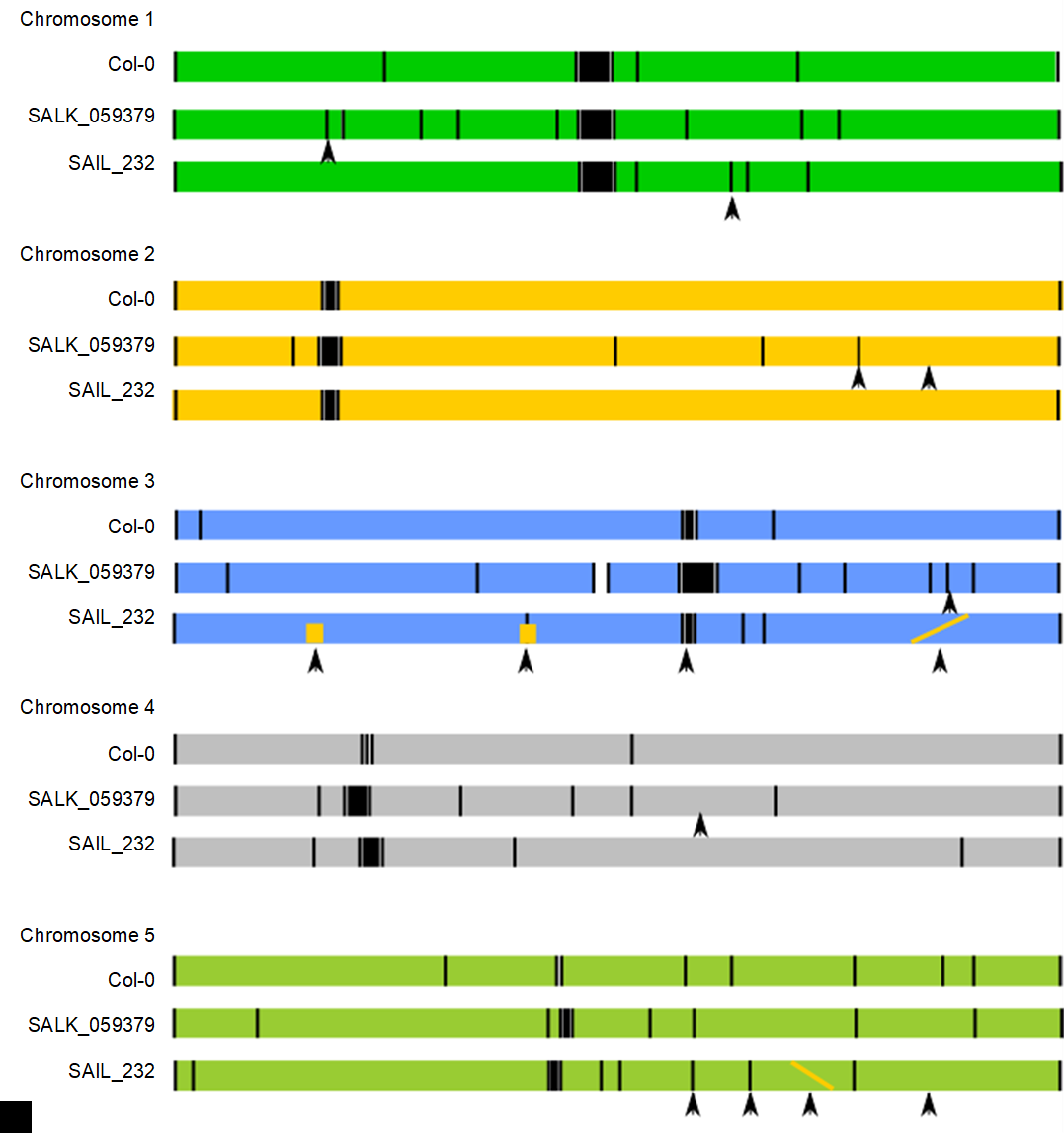

Supplement: S1 Fig — Uninterrupted colored blocks indicate contig length, black bars indicate the start and end of contigs, black boxes indicate centromere gaps. Arrowheads represent T-DNA insertion sites; orange lines and boxes indicate sites of translocations. Drawn to scale for each chromosome individually. (TIF) [file pgen.1007819.s011.tif]

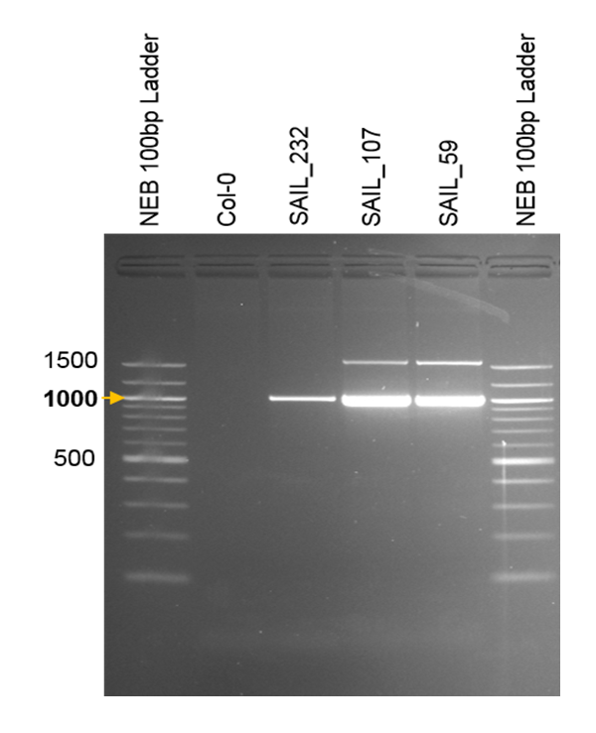

Supplement: S2 Fig — Two additional SAIL lines were chosen at random and genotyped along with SAIL_232. The primers were designed to amplify 1000bp spanning the junction between the genomic and inverted DNA sequence (based on de novo reads from ONT sequencing). The inversion on chromosome 1 was seen in all SAIL lines tested, unlike the other chromosomal architecture disruptions found. Thus, it can be concluded that this inversion is part of the Col-3 background from which SAIL lines are derived. The bands at 1500bp in the SAIL_107 and SAIL_59 lines are off-target amplification products. (TIF) [file pgen.1007819.s012.tif]

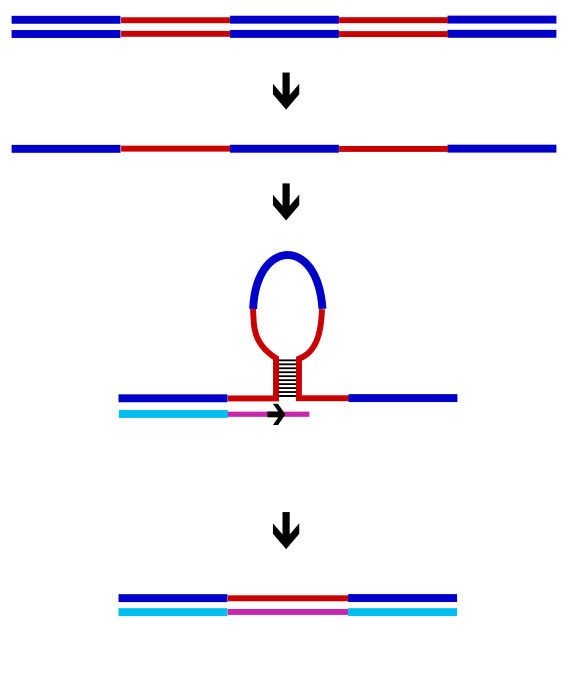

Supplement: S3 Fig — (a) A double strand chromosome break leads to (b) two multi-copy T-DNA strand insertions in opposing directions. The exact structure and length of the inserted sequence is unknown, as indicated by question marks. (c) SALK_chr2:18Mb insertion features two individual double strand breaks, around 5 kb apart. High homology between the T-DNA strands as well as the hairpin forming original DNA piece created a secondary structure (d), that was potentially excised (e) and resulted in the deletion of the ~5kb chromosomal fragment, as shown in main text Fig 2. Arrowheads on the red T-DNA strand show direction. The blue line represents double stranded DNA. (TIF) [file pgen.1007819.s013.tif]

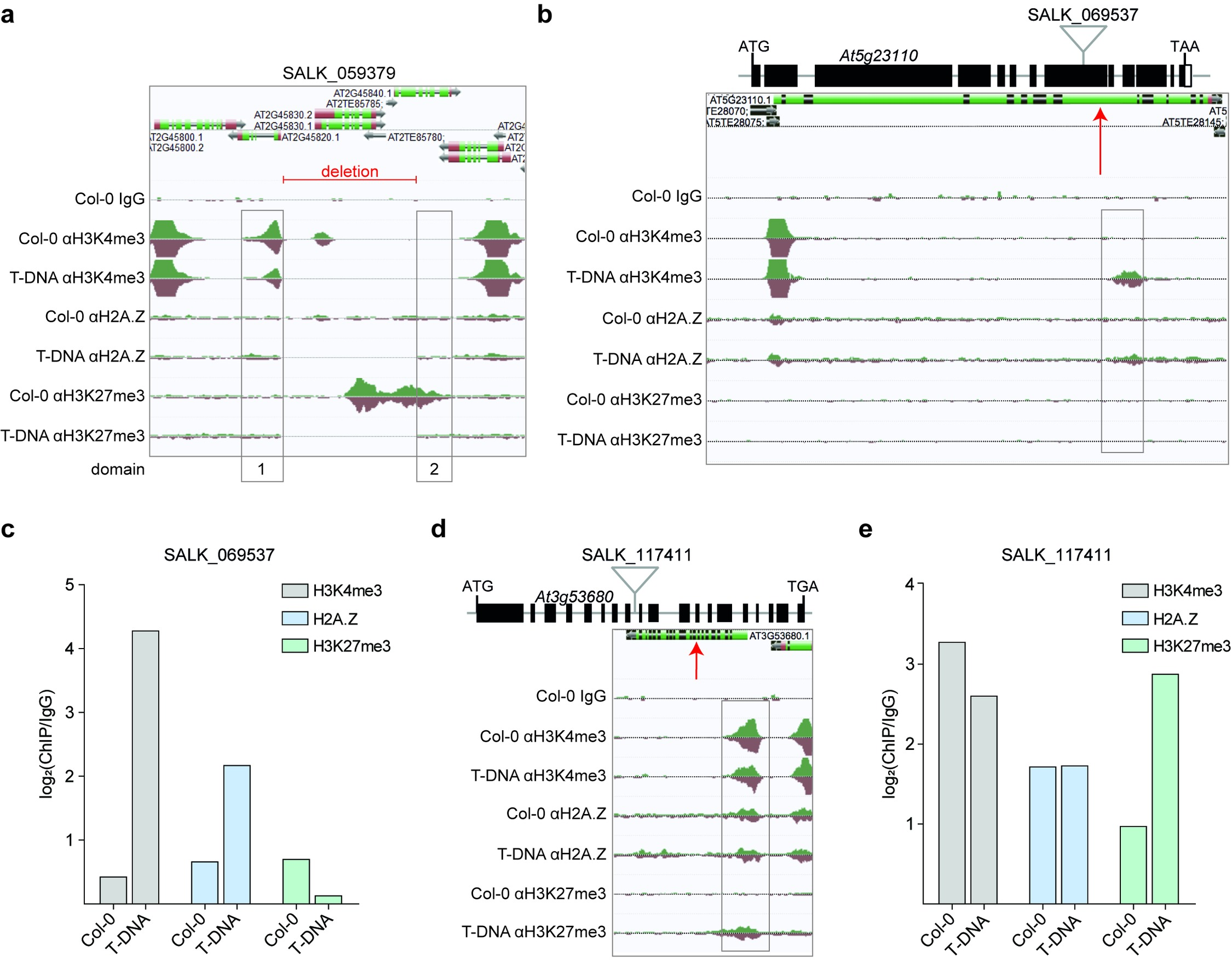

Supplement: S4 Fig — We applied blastn searches of all unassembled ONT reads against the utilized vector sequence, and subsequently the TAIR10 reference genome. This identified reads such as the depicted, that confirm insertion events (like SALK_059379 on Chr 4 at 10.4 Mb) not present in the assembly or BNG maps. Our blastn strategy identified chromosomal sequence (yellow), and individual alignments with the pROK plasmid sequence revealed T-strand (blue) and vector backbone (green) sequence. The pink plasmid sequence within the vector backbone shows an internal breakpoint, which was likely caused by multiple independent insertion events within the same region. Percent identity of the raw read stretch to the reference sequence are listed within the annotations. (TIF) [file pgen.1007819.s014.tif]

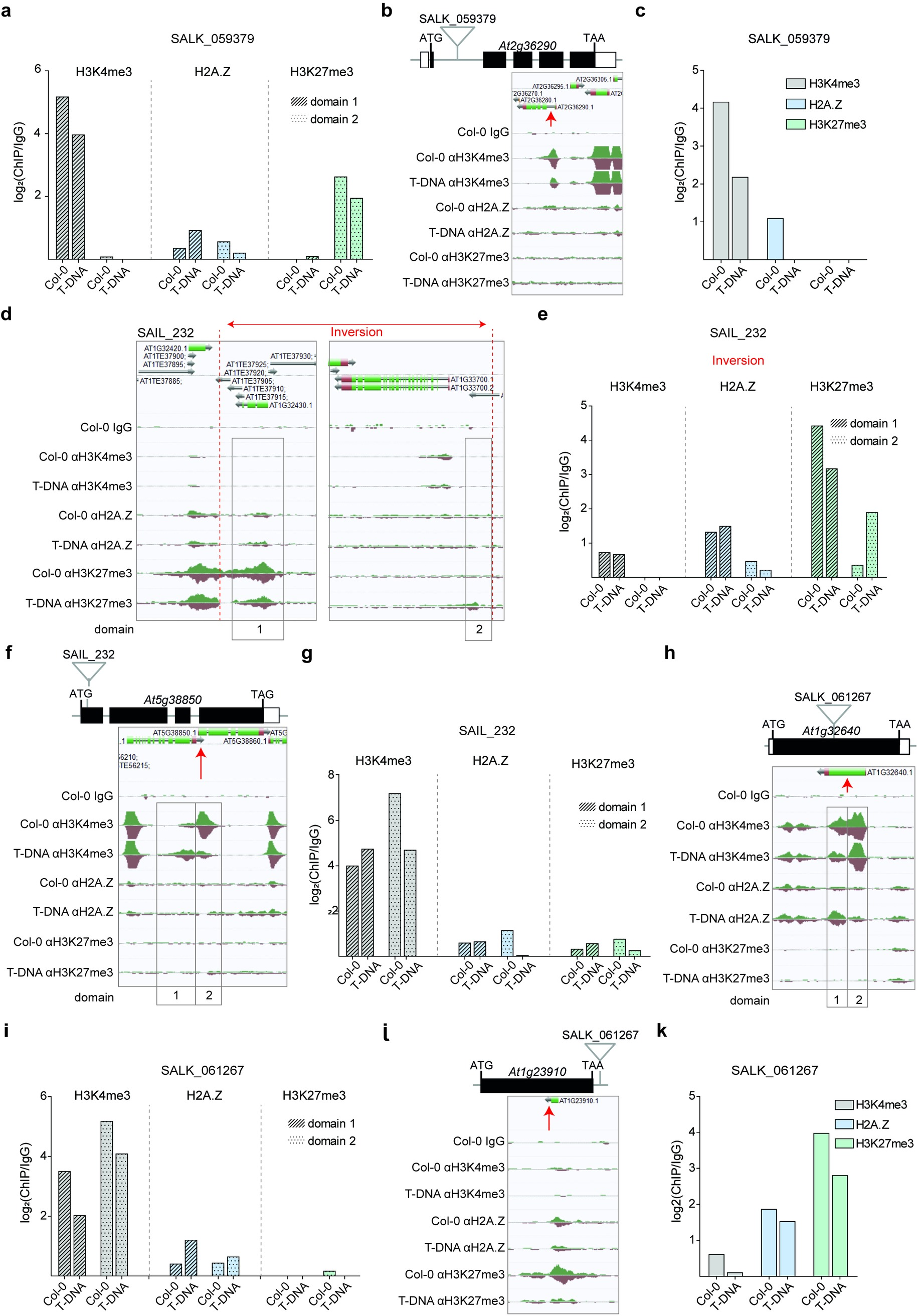

Supplement: S5 Fig — (a) Quantification of H3K4me3, H2A.Z and H3K27me3 at the two domains next to the T-DNA deletion in SALK_059379 is shown. (b), (c) Impact of the T-DNA integration on the local chromatin environment at At2g36280 in SALK_059379 seedlings. Visualization (b) and quantification (c) of H3K4me3, H2A.Z and H3K27me3 occupancy around the T-DNA insertion site in Col-0 and SALK_059379 seedlings is shown as well as the schematic illustration of the gene structure of At2g36280 (b) including the approximate localization of T-DNA insertion. (d), (e) Visualization (d) and quantification (e) of H3K4me3, H2A.Z and H3K27me3 occupancy at flanking regions of the SAIL specific WT inversion on chromosome 1. The corresponding flanking regions in Col-0 were indicated as domain 1 and 2. (f), (g), Impact of the T-DNA integration on the local chromatin environment at At5g38850 in SAIL_232 seedlings. Visualization (f) and quantification (g) of H3K4me3, H2A.Z and H3K27me3 occupancy around the T-DNA insertion site in Col-0 and SAIL_232 seedlings is shown. Schematic illustration of the gene structure of At5G38850 (f) indicates the approximate localization of the T-DNA insertion. (h), (i) Impact of the T-DNA integration on the local chromatin environment at At1g32640 in SALK_061267 seedlings. Visualization (h) and quantification (i) of H3K4me3, H2A.Z and H3K27me3 occupancy around the T-DNA insertion site in Col-0 and SALK_061267 seedlings is shown as well as the schematic illustration of the gene structure of At1g32640 (h) including the approximate localization of T-DNA insertion. (j), (k) Impact of the T-DNA integration on the local chromatin environment at At1g23910 in SALK_061267 seedlings. Visualization (j) and quantification (k) of H3K4me3, H2A.Z and H3K27me3 occupancy around the T-DNA insertion site in Col-0 and SALK_061267 seedlings is shown. Schematic illustration of the gene structure of At1g23910 (j) indicates the approximate localization of the T-DNA insertion. A red arrow marks the approx [file pgen.1007819.s015.tif]

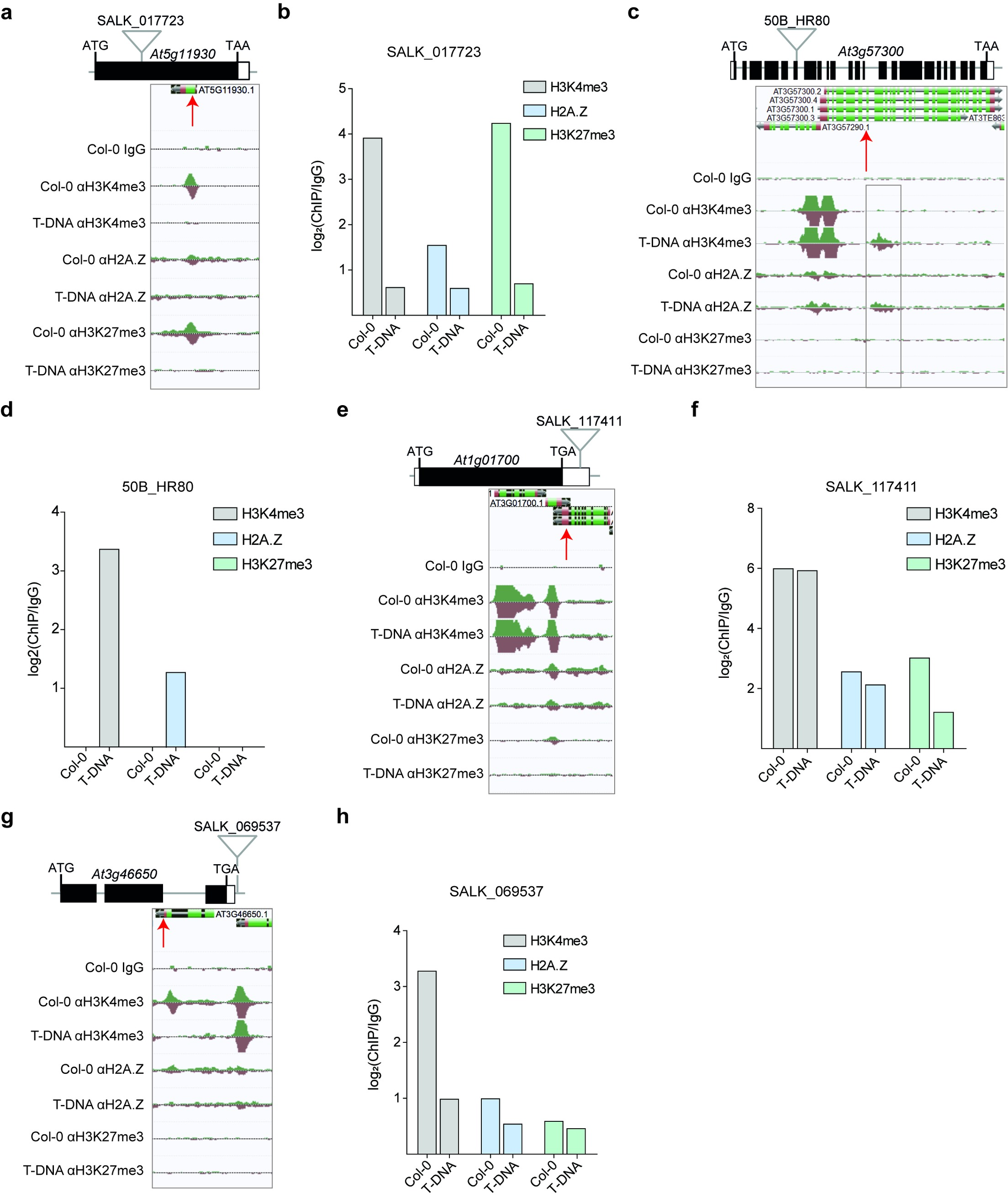

Supplement: S6 Fig — (a), (b) Impact of the T-DNA integration on the local chromatin environment at At5g11930 in SALK_017723 seedlings. Visualization (a) and quantification (b) of H3K4me3, H2A.Z and H3K27me3 occupancy around the T-DNA insertion site in Col-0 and SALK_017723 seedlings is shown as well as the schematic illustration of the gene structure of At5g11930 (a) including the approximate localization of T-DNA insertion. (c) Genome browser visualizes the T-DNA-induced de novo trimethylation of H3K4 and incorporation of H2A.Z at in 50B_HR80 seedlings. The new T-DNA-induced domain within the gene body of At3g57300 is indicated as well as a schematic illustration of the gene structure of At3g57300 including the approximate localization of the corresponding T-DNA insertion. (d) Quantification of the new H3K4me3 and H2A.Z domain within the gene body of At3g57300 is shown. (e), (f) Impact of the T-DNA integration on the local chromatin environment at At1g01700 in SALK_117411 seedlings. Visualization (e) and quantification (f) of H3K4me3, H2A.Z and H3K27me3 occupancy around the T-DNA insertion site in Col-0 and SALK_117411 seedlings is shown as well as the schematic illustration of the gene structure of At1g01700 (e) including the approximate localization of T-DNA insertion. (g), (h) T-DNA integration impacts the local chromatin environment at At3g46650 in SALK_069537 seedlings. Visualization (g) and quantification (h) of H3K4me3, H2A.Z and H3K27me3 occupancy around the T-DNA insertion site in Col-0 and SALK_069537 seedlings is shown. Schematic illustration of the gene structure of At3g46650 (g) indicates the approximate localization of the T-DNA insertion. All ChIP-seq data was visualized with the AnnoJ genome browser. A red arrow marks the approximate location of the T-DNA integration site in the Annoj genome browser screenshots. H3K4me3, H2A.Z and H3K27me3 occupancy was profiled with ChIP-seq whereby the Col-0 IgG sample serves as a negative control. All shown AnnoJ genome browser trac [file pgen.1007819.s016.tif]
